# Bone Alkaline Phosphatase and Tartrate-Resistant Acid Phosphatase: Potential Co-regulators of Bone Mineralization

**DOI:** 10.1007/s00223-017-0259-2

**Published:** 2017-03-16

**Authors:** Cecilia Halling Linder, Barbro Ek-Rylander, Michael Krumpel, Maria Norgård, Sonoko Narisawa, José Luis Millán, Göran Andersson, Per Magnusson

**Affiliations:** 1 Department of Clinical Chemistry and Department of Clinical and Experimental Medicine, Linköping University, 581 85 Linköping, Sweden; 2 Division of Pathology, Department of Laboratory Medicine, Karolinska Institutet, 141 86 Huddinge, Sweden; 3 Sanford Children’s Health Research Center, Sanford Burnham Prebys Medical Discovery Institute, La Jolla, CA 92037 USA

**Keywords:** Bone, Dephosphorylation, Hydroxyapatite, Inorganic pyrophosphate, Mineralization, Osteopontin

## Abstract

Phosphorylated osteopontin (OPN) inhibits hydroxyapatite crystal formation and growth, and bone alkaline phosphatase (BALP) promotes extracellular mineralization via the release of inorganic phosphate from the mineralization inhibitor inorganic pyrophosphate (PPi). Tartrate-resistant acid phosphatase (TRAP), produced by osteoclasts, osteoblasts, and osteocytes, exhibits potent phosphatase activity towards OPN; however, its potential capacity as a regulator of mineralization has not previously been addressed. We compared the efficiency of BALP and TRAP towards the endogenous substrates for BALP, i.e., PPi and pyridoxal 5′-phosphate (PLP), and their impact on mineralization in vitro via dephosphorylation of bovine milk OPN. TRAP showed higher phosphatase activity towards phosphorylated OPN and PPi compared to BALP, whereas the activity of TRAP and BALP towards PLP was comparable. Bovine milk OPN could be completely dephosphorylated by TRAP, liberating all its 28 phosphates, whereas BALP dephosphorylated at most 10 phosphates. OPN, dephosphorylated by either BALP or TRAP, showed a partially or completely attenuated phosphorylation-dependent inhibitory capacity, respectively, compared to native OPN on the formation of mineralized nodules. Thus, there are phosphorylations in OPN important for inhibition of mineralization that are removed by TRAP but not by BALP. In conclusion, our data indicate that both BALP and TRAP can alleviate the inhibitory effect of OPN on mineralization, suggesting a potential role for TRAP in skeletal mineralization. Further studies are warranted to explore the possible physiological relevance of TRAP in bone mineralization.

## Introduction

Skeletal remodeling and maintenance is an ongoing process with continuous resorption of bone by osteoclasts and formation of new bone by osteoblasts. Bone tissue is made of collagen fibers that form a scaffold where calcium and phosphate, mainly in the form of crystalline hydroxyapatite (HA), are deposited [1]. Mineralization is initiated by the accumulation of calcium and inorganic phosphate, followed by crystal growth [2, 3]. To obtain a normal mineral deposition rate during bone remodeling, the mineralization process is controlled by several molecules that either inhibit or promote the growth of HA crystals [4, 5].

Osteopontin (OPN), an important regulator of HA crystal formation and growth, is a multifunctional, highly phosphorylated protein expressed at high levels in mineralizing tissue such as bones and teeth, but also in some soft tissues and body fluids [4, 6–8]. OPN belongs to the small integrin-binding ligand N-linked glycoprotein (SIBLING) family and is a characteristic intrinsically disordered protein with structures that are highly flexible [4, 9]. This flexibility enables OPN to rapidly interact with proteins, e.g., collagen, as well as hydroxyapatite crystals [10]. Different posttranslational modifications such as glycosylation, sulfation, transglutamination, and phosphorylation influence the functional properties of OPN [5, 7, 11]. The secreted Golgi casein kinase Fam20C is highly expressed in mineralized tissues and appears to be the enzyme regulating HA formation by phosphorylation of SIBLING proteins. Mutations in Fam20C cause an osteosclerotic bone dysplasia in humans known as Raine syndrome [12]. The number of phosphorylations varies between OPN isolated from different species and sources. The reason for the occurrence of different degrees of phosphorylation is not fully understood but may be related to the different actions of phosphatases on OPN. Highly phosphorylated OPN forms the so-called calcium phosphate nanoclusters from amorphous calcium phosphate precipitates, which is of physiological importance since this delays HA crystallization [13]. Phosphorylation and dephosphorylation of OPN appear to control several of its biological functions, such as cell adhesion and migration [14]. Milk OPN has 28–36 phosphorylations and OPN isolated from bone has 12–13 phosphorylations on average [7, 11, 15, 16], a difference possibly related to the action of extracellular phosphatases such as bone alkaline phosphatase (BALP) and tartrate-resistant acid phosphatase (TRAP).

TRAP, also referred to as type 5 acid phosphatase/AcP 5, is highly expressed in osteoclasts [17] but is also expressed in osteoblasts and osteocytes [18, 19]. TRAP is synthesized as a monomer with low enzyme activity; however, the monomer (TRAP 5a) can be converted into a dimer (TRAP 5b) with high enzymatic activity by posttranslational proteolytic processing [20–22]. TRAP 5b exerts phosphatase activity towards OPN and bone sialoprotein [23]; however, its potential capacity as a regulator of mineralization has not previously been addressed.

Alkaline phosphatase (ALP) is a glycoprotein and functions as an ectoenzyme attached to the outer surface of cells and matrix vesicles. In humans, there are four genes encoding the ALP isozymes, i.e., intestinal ALP (IALP), placental ALP, germ cell ALP, and tissue-nonspecific ALP (TNALP) expressed in bone (as BALP), liver, and kidney [24]. Studies of hypophosphatasia, a rare inborn-error-of-metabolism, caused by missense mutations within the TNALP gene (*ALPL*), have provided evidence for an important role for ALP in the development and mineralization of bone [25]. Hypophosphatasia in TNALP knockout mice results in increased inorganic pyrophosphate (PPi) concentrations and a concomitant increase in OPN phosphorylation levels; the combined effect of these molecules leads to hypomineralization [26].

A model to explain the differential roles of the two phosphatases BALP and TRAP is missing and their possible functional interplay remains to be explored. In this study, we hypothesized that BALP and TRAP might substitute for each other as regulators of mineralization. The kinetic properties of TRAP towards the known endogenous substrates of BALP, i.e., PPi and pyridoxal 5′-phosphate (PLP) [25, 27], were investigated in order to explore the possible relevance of TRAP in skeletal mineralization. Furthermore, it was also investigated how BALP and TRAP can act as regulators of mineralization by dephosphorylating the mineralization inhibitor OPN.

## Materials and Methods

### Materials

All reagents were obtained from Sigma-Aldrich (St. Louis, MO, USA) if not stated otherwise. BALP, extracted from human bone tissue, was obtained from Calzyme Laboratories Inc. (San Luis Obispo, CA, USA; Cat#: 124A0001) and IALP from bovine intestinal mucosa (Sigma-Aldrich; Cat#: A2356). Purification of recombinant human TRAP 5a from concentrated Baculovirus-infected *Spodoptera frugiperda* (Sf9) insect cell culture supernatant (obtained from GenScript USA Inc., Piscataway, NJ, USA) was performed according to a previously published protocol [28]. For proteolytic cleavage of TRAP 5a to enzymatically active TRAP 5b, human liver cathepsin L (Merck Millipore, Darmstadt, Germany) was used according to Krumpel et al. [28]. Bovine milk OPN was purified according to Bayless et al. [29] as modified by Ljusberg et al. [20] In brief, OPN was purified from 1 L of raw bovine milk, after the addition of a protease inhibitor cocktail (Roche Diagnostics Scandinavia AB, Bromma, Sweden; Cat#:1697498), using one DEAE Sepharose Fast Flow column (GE Healthcare Bio-Sciences AB, Uppsala, Sweden; Cat#:17-0709-01) and two consecutive Phenyl Sepharose Fast Flow columns (GE Healthcare Bio-Sciences AB; Cat#:17-0973-05). The identity and purity of the isolated OPN were confirmed by SDS-PAGE with silver staining and by amino-terminal sequence analysis, and the concentration was determined by total amino acid analysis.

### Determination of Kinetic Properties for BALP and TRAP

Kinetic properties were evaluated for both BALP and TRAP 5b using the endogenous substrates for BALP, i.e., PPi and PLP, as well as the synthetic substrate p-nitrophenylphosphate (pNPP). The kinetic properties for TRAP 5b were determined in a buffer with a final concentration of 0.1 M sodium acetate at pH 5.8, 0.15 M KCl, 0.1% (v/v) Triton X-100, 10 mM disodium tartrate, 1 mM ascorbic acid, and 0.1 mM Fe(NH_4_)_2_(SO_4_) (TRAP buffer). The kinetic measurements of BALP were carried out in a buffer with a final concentration of 0.2 mM (NH_4_)_2_CO_3_ at pH 8.5, 2 mM MgCl_2_, 40 µM zinc acetate, and 10 µg/mL E-64 (ALP buffer). TRAP 5b was diluted in TRAP buffer to a final concentration of 0.03 ng/µL for the measurements with pNPP and PPi, and 0.3 ng/µL for PLP. BALP was diluted in ALP buffer to a final concentration of 0.04 µg/µL for all three substrates. All measurements were carried out in 96-well plates and incubated at 37 °C for 30 min. For the kinetic measurements, 25 µL of the substrate was added to the final concentrations of 0.05 mM to 10 mM together with 25 µL TRAP 5b or BALP solution and 50 µL buffer (TRAP or ALP buffer). After 30 min, 50 µL stop solution was added to each sample, 0.5 M NaOH for the pNPP reaction and 0.2 M Na_2_MoO_4_ for PPi and PLP. For pNPP, the amount of formed p-nitrophenol was measured by absorbance at 405 nm, and for PPi and PLP the amount of liberated free phosphate was determined using the Biomol Green Reagent (Enzo Life Sciences Inc., Farmingdale, NY, USA).

The kinetic parameters, maximum reaction velocity (*V*_max_), and the Michaelis constant (*K*_m_) were determined from a Lineweaver–Burk plot where the substrate concentration was plotted against the specific enzyme activity.

### Dephosphorylation of OPN by BALP, IALP, and TRAP

For time curve analyses, the enzyme activity needed for maximal dephosphorylation of 10 µg bovine milk OPN, after 24-h incubation at 37 °C, was chosen for each phosphatase. PNPP equivalents corresponding to 5 mU TRAP 5b, 20 mU BALP, and 20 mU IALP were incubated with 10 µg bovine milk OPN in siliconized Eppendorf tubes. Dephosphorylation with TRAP was carried out in a buffer containing 0.1 M sodium acetate at pH 5.0, 0.15 M KCl, 10 mM disodium tartrate, 1 mM ascorbic acid, and 0.1 mM Fe(NH_4_)_2_(SO_4_), and stopped at different time points, 0–24 h, with 10 mM Na_2_MoO_4_. Dephosphorylation with BALP and IALP was performed in a buffer containing 200 mM (NH_4_)_2_CO_3_ at pH 8.5, 2 mM MgCl_2_, and 40 µM zinc acetate, and stopped at different time points with 20 mM EDTA. Liberated phosphate was determined at each time point by the addition of 100 µL of Biomol Green Reagent to 20 µL dephosphorylated OPN and incubated for 20 min at room temperature.

Dephosphorylation of OPN was also investigated with different quantities of TRAP 5b (0.5, 1.25, 2.5, and 5 mU), and for BALP and IALP (5, 10, 20, and 40 mU). The amount of liberated free phosphate was measured with the Biomol Green Reagent after incubation for 24 h.

### In Vitro Mineralization

Human osteoblast-like SaOS-2 cells (ATCC, American Type Culture Collection, Manassas, VA, USA) were grown in 96-well black-walled plates in Dulbecco’s modified Eagle’s medium—low glucose, supplemented with 1% fetal calf serum, 1% penicillin/streptomycin, and 40 U/mL nystatin, at 37 °C with 95% humidity and 5% CO_2_.

Mineralization was initiated 24 h after plating out the cells. The medium was replaced with a fresh medium supplemented with 2 mM β-glycerophosphate and 50 µg/mL ascorbic acid in order to initiate mineralization. Fully phosphorylated OPN and partially dephosphorylated OPN (by BALP or TRAP) were added to a final concentration of 0.1 µg/mL. Cells were cultured for 5 days after the initiation of mineralization and the medium was changed on days 2 and 4. The amount of mineral was quantified using the OsteoImage Mineralization Assay (Lonza Walkersville, Inc., Walkersville, MD, USA), which specifically binds to HA nodules. This assay is, unlike typical histochemical methods such as von Kossa and Alizarin Red staining, HA-specific [30, 31]. The OsteoImage Mineralization Assay is an in vitro assay that can quantitate bone cell mineralization and is based on the specific binding of the fluorescent OsteoImage staining reagent to the HA portion of the bone-like nodules deposited by cells. The medium was removed after 5 days and the cells were fixed with 99% ethanol and incubated with the fluorescent OsteoImage reagent. Fluorescence was determined using a Fluoroskan Ascent FL fluorescent microplate reader (Thermo Fisher Scientific, Vantaa, Finland) with excitation/emission set at 485/538 nm. The measured fluorescence is proportional to the amount of HA present in the culture.

Images of mineralizing cells were captured with a 10× objective (NA 0.3) on a Zeiss Axio Observer Z1 with an AxioCam MRm camera (Carl Zeiss MicroImaging, Thornwood, NY, USA) and excitation/emission set at 485/538 nm. Images of the cells grown were captured: (i) without β-glycerophosphate and ascorbic acid; (ii) with 2 mM β-glycerophosphate and 50 µg/mL ascorbic acid; (iii) and with 2 mM β-glycerophosphate, 50 µg/mL ascorbic acid, and OPN (fully phosphorylated).

### Statistical Analysis

Data were analyzed using the Excel software (Microsoft, Redmond, WA, USA). Results are presented as mean ± standard deviation (SD). Statistical analyses were performed using unpaired two-tailed Student’s *t* test for comparisons between two groups, and ANOVA was used to test for differences involving more than two groups. For all statistical tests, a difference was considered significant at *P* < 0.05.

## Results

### Kinetic Properties for BALP and TRAP

Both BALP and TRAP 5b displayed catalytic activity for the three substrates PPi, PLP, and pNPP (Table 1). The enzymatic activities at their respective pH optima (i.e., BALP, pH 8.5; TRAP 5b, pH 5.8) were significantly higher for TRAP in comparison with BALP for all substrates, but were particularly pronounced for pNPP and PPi. No significant differences in *K*_m_ for the different substrates for BALP and TRAP were noted. The *V*_max_ values were 4300-fold and 730-fold higher for pNPP and PPi, respectively, for TRAP in comparison with BALP. For the substrate PLP, *V*_max_ was fourfold higher for TRAP in comparison with BALP.

**Table 1:** Kinetic properties for the endogenous substrates (for BALP), PPi and PLP, and the synthetic substrate pNPP

	TRAP	BALP
*V*_max_ (mU/mg)		
PPi	22,000 ± 2160	30 ± 13**
PLP	390 ± 7	90 ± 16*
pNPP	774,000 ± 160,000	180 ± 9*
*K*_m_ (mM)		
PPi	1.49 ± 0.10	2.45 ± 1.38
PLP	0.34 ± 0.28	0.12 ± 0.04
pNPP	3.00 ± 1.60	0.31 ± 0.01

Results are expressed as mean ± SD of three independent experiments

* *P* < 0.05, ** *P* < 0.005, in comparison with TRAP

### Dephosphorylation of OPN by BALP and TRAP

**Fig. 1:**
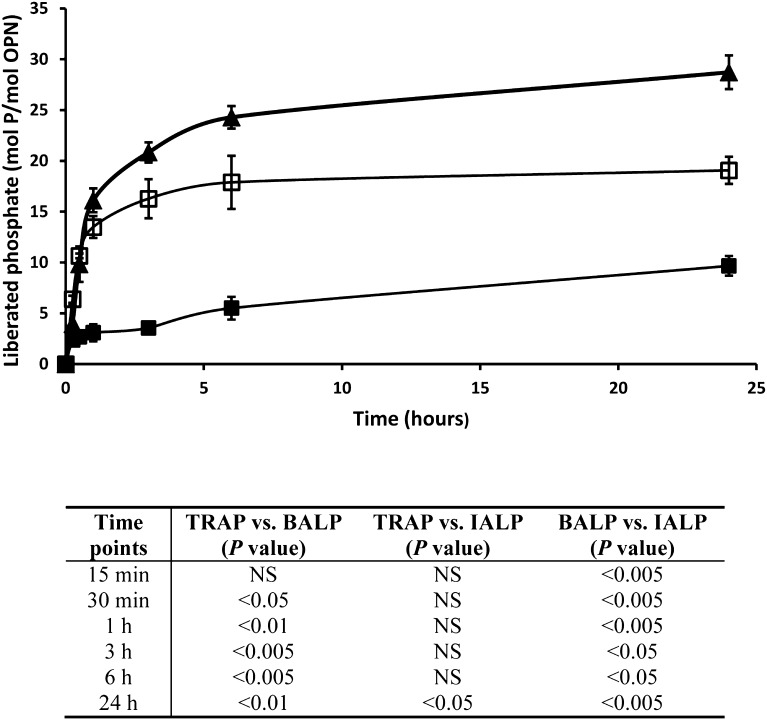
Dephosphorylation of OPN by TRAP, BALP, and IALP. OPN was completely dephosphorylated (28 free phosphates liberated) after 24-h incubation with 5 mU of TRAP (*filled triangle*). During the same time period, 20 mU of BALP (*filled square*) cleaved off 10 free phosphates and 20 mU IALP (*open square*) liberated 20 free phosphates from OPN. All experiments were run in triplicate. Statistical comparisons were made between TRAP, BALP, and IALP at each time point (*NS* not significant)

**Fig. 2:**
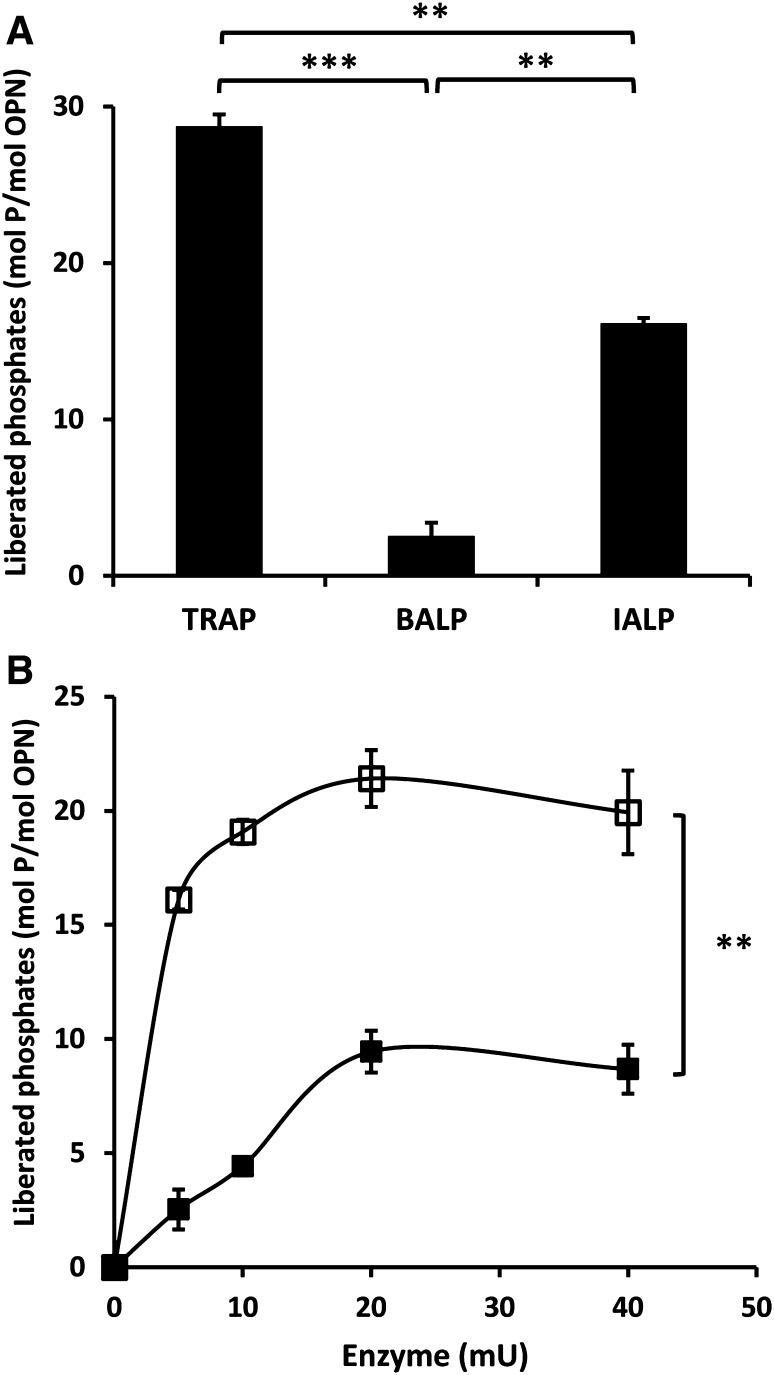
**A** Differences between TRAP, BALP, and IALP regarding the dephosphorylation efficiency on OPN. These results demonstrate the amount of liberated free phosphate cleaved off from OPN after 24-h incubation with 5 mU of TRAP, BALP, and IALP. **B** Maximum amount of phosphate liberated from the OPN molecule after 24 h of incubation with different concentrations of BALP (*filled square*) and IALP (*open square*). Statistical comparisons were made between the maximum values of liberated free phosphate for each enzyme after 24-h incubation of each enzyme. Results are presented as mean ± SD of three independent experiments. ** *P* < 0.01, *** *P* < 0.001

OPN purified from bovine milk contains 28 phosphorylations on 27 serine residues and 1 threonine residue [16]. When treated with 5 mU TRAP, OPN was completely dephosphorylated after 24 h, whereas 20 mU BALP dephosphorylated OPN only partially with ten phosphates being removed (36%) (Fig. 1). IALP is often used to dephosphorylate OPN in experimental studies [32]; however, after incubation with 20 mU IALP, only 20 phosphates (71%) were removed after 24 h (Fig. 1). Raising the amounts of BALP and IALP to 40 mU did not increase the amount of liberated free phosphate from OPN at 24 h (Fig. 2).

**Fig. 3:**
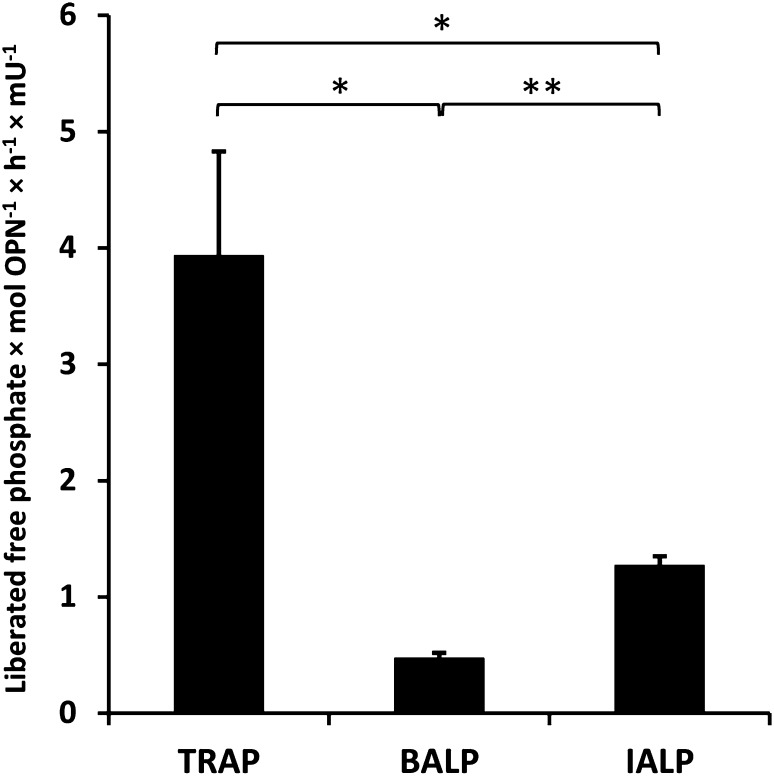
Dephosphorylation of OPN with TRAP, BALP, and IALP with respect to liberated free phosphate per mol OPN calculated per hour and mU enzyme. * *P* < 0.05, ** *P* < 0.01

Comparison of the dephosphorylation rates of TRAP, BALP, and IALP demonstrated that TRAP can liberate 3.9 free phosphates per OPN molecule per hour and mU, whereas BALP and IALP liberate 0.5 and 1.3 free phosphates, respectively (Fig. 3).

### In Vitro Mineralization

**Fig. 4:**
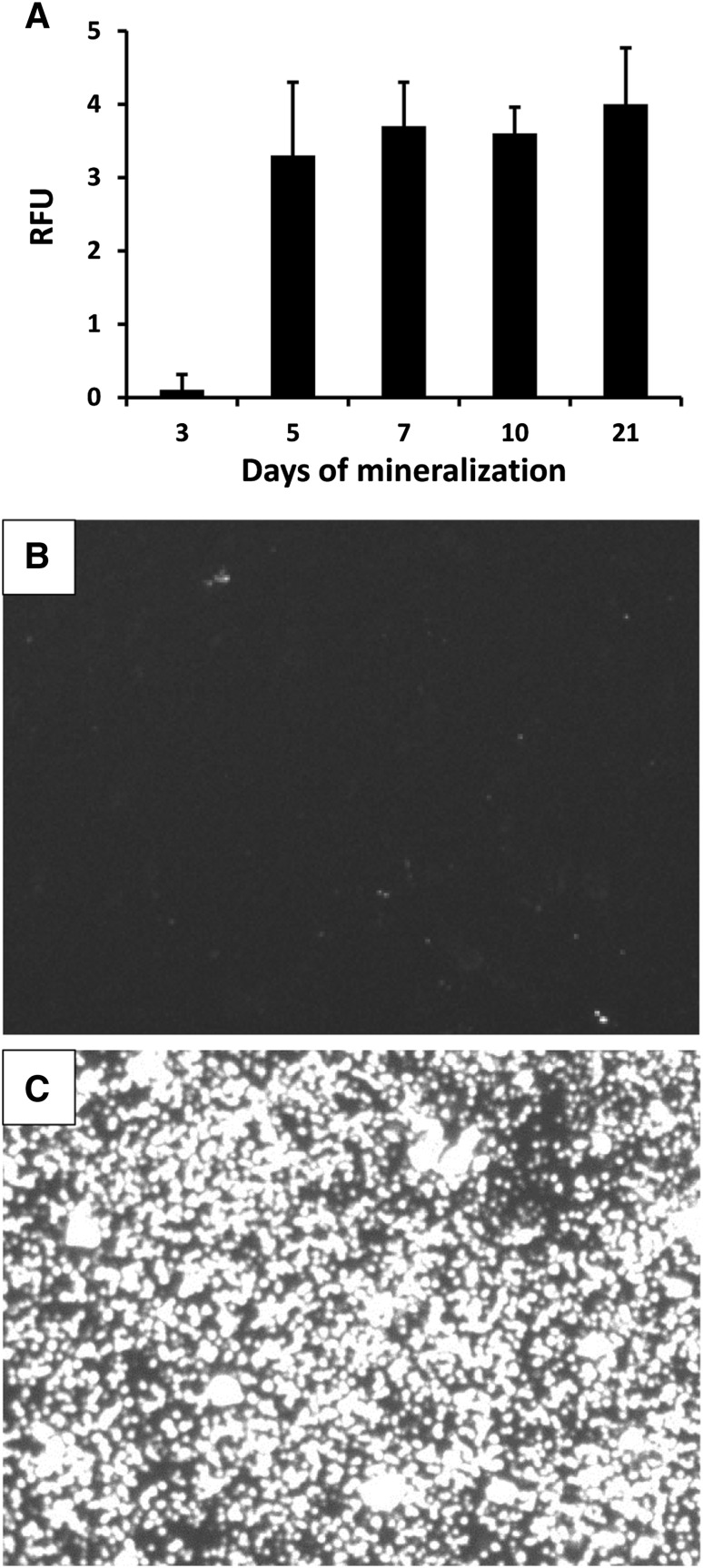
**A** Osteoblast-like SaOS-2 cells cultured for 3, 5, 7, 10, and 21 days after initiation of mineralization (without OPN). Cells were stained with the OsteoImage Mineralization Assay, which specifically detects the HA portion of bone-like nodules deposited by cells. Fluorescence was measured in relative fluorescence units (RFU) and is proportional to the amount of HA present in the culture. Results are presented as mean ± SD of eight samples. **B** Image of cells cultured for 10 days without mineralization medium. **C** Image of cells cultured for 10 days with mineralization medium (without OPN)

**Fig. 5:**
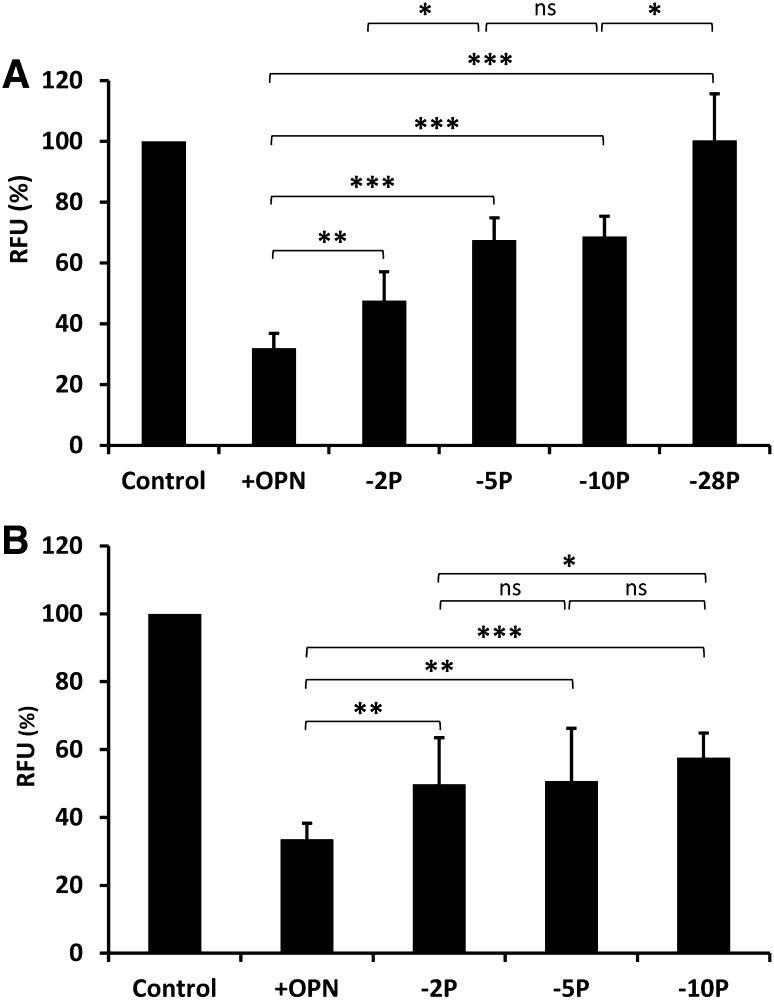
Amount of mineralization in osteoblast-like SaOS-2 cells 5 days after initiation of mineralization. Cells grown with mineralization medium but without OPN were defined as controls and considered to be fully mineralized, and set to 100% relative fluorescence units (RFU). The other bars are expressed as percentages of 100% RFU. +OPN indicates the addition of OPN (fully phosphorylated), and −2P, −5P, −10P, and −28P indicate the number of phosphates cleaved off (dephosphorylated OPN). OPN, fully phosphorylated and dephosphorylated, was added at a final concentration of 0.1 µg/mL. Results are presented as mean ± SD of three independent experiments with eight samples in each experiment. **A** OPN fully phosphorylated and dephosphorylated by TRAP. **B** OPN fully phosphorylated and dephosphorylated by BALP. * *P* < 0.05, ** *P* < 0.01, *** *P* < 0.005, *ns* not significant

In the mineralization model, the accumulation of HA deposits from osteoblast-like SaOS-2 cells (stimulated by mineralization media including β-glycerophosphate and ascorbic acid) was detected and quantitated by fluorescent staining (Fig. 4). The highest amount of HA deposition was observed after the first 5 days of cultivation, and longer cultivation time did not further increase the amount of HA deposition (Fig. 4A). The inhibitory effect of fully phosphorylated OPN and OPN dephosphorylated with TRAP and BALP was studied by measuring the amount of HA produced after initiation of mineralization. Cells grown only with mineralization medium, without OPN, were defined as controls and considered to be fully mineralized and set to 100%. Fully phosphorylated OPN decreased the amount of produced HA by 67% (Fig. 5). Dephosphorylation of OPN with TRAP gradually decreased the inhibitory effect of OPN the more phosphate that was cleaved off. Fully dephosphorylated OPN (i.e., −28 phosphates by TRAP) had no inhibitory effect on the mineralization process in this in vitro model (Fig. 5A). BALP, removing two phosphates per mol OPN, decreased the inhibitory capacity of OPN by approximately one-third to the same level as observed when removing five phosphates. Removing ten phosphates reduced the inhibitory capacity in comparison with OPN with only two phosphates being removed (Fig. 5B).

## Discussion

Besides being highly expressed in osteoclasts, TRAP is also expressed in osteoblasts and osteocytes [18, 19]; however, the potential capacity for TRAP as a regulator of mineralization, with or without a functional interplay with BALP, has not been addressed. The present work demonstrates that TRAP can completely dephosphorylate the mineralization inhibitor OPN (–28 phosphates), whereas BALP and IALP dephosphorylate OPN by 36% (–10 phosphates) and 71% (–20 phosphates), respectively. In a previous study by Ek-Rylander et al. [33], no dephosphorylation of OPN was observed with TNALP from bovine kidney. However, in the present study, using dose–response and time course assays, we observed a significant OPN phosphatase activity of TNALP, purified from bone, although at a lesser extent in comparison with TRAP. TRAP also displayed a much higher phosphatase activity towards PPi and pNPP in comparison with BALP, whereas the activity of TRAP and BALP towards PLP was similar. To our knowledge, there is only one previous study that has investigated the kinetic properties of TRAP towards PPi, but none regarding PLP. Lam et al. [34] reported that TRAP has similar reactivity towards pNPP and PPi. Both BALP and TRAP are present in osteoblasts and exhibit significant activity against the mineralization inhibitor PPi, which indicates that both these phosphatases could potentially participate in the regulation of the intricate process of mineralization.

Unlike BALP, TRAP is expressed in osteocytes [19, 35]. There are data suggesting that osteocytes remodel its lacunae and canaliculae [36, 37], which involves similar mechanisms that osteoclasts use for resorbing bone (i.e., acidification, demineralization, and collagen degradation). The subsequent formation process in the osteocyte lacuna remains elusive; however, we suggest that this process could be mediated by TRAP, instead of BALP, in controlling the inhibitory effect of PPi and/or OPN.

OPN is expressed in several tissues, including bone matrix, and is involved in numerous biological processes. The degree of phosphorylation of OPN is of significant importance for its functional properties. For example, dephosphorylation of OPN by TRAP influences the migration and attachment of osteoclasts [14], and phosphorylated OPN peptides have previously been shown to inhibit HA formation in vitro, while the same peptides without phosphate lacked inhibitory effect on mineralization [38]. The current study shows that the inhibitory effect of OPN on mineralization was significantly influenced by the degree of phosphorylation in cultures with osteoblast-like cells. TRAP dephosphorylated OPN completely, which totally eliminated the inhibitory action of OPN on mineralization, while BALP only removed 36% of the phosphates, which resulted in a partial restoration of mineralization. In contrast, Hunter et al. [39] were able to remove 84% of bound phosphate from OPN with the use of ALP coupled to agarose beads. The source of ALP immobilized on these agarose beads is commonly calf IALP in very high concentrations because IALP is a rather inexpensive and widespread phosphatase. In a cell-free system, this approach of dephosphorylation reduced the de novo formation of HA by more than 40-fold [39]. The same method with IALP-coupled agarose beads to dephosphorylate OPN was applied by Jono et al. [32], who demonstrated that bacterium-derived recombinant OPN (with 20 phosphates) phosphorylated by casein kinase II inhibited human smooth muscle cell culture calcification, while dephosphorylation of the same OPN did not. In this study, we demonstrate that BALP and IALP differ significantly in their dephosphorylating properties. Hence, the results and conclusions from investigating both skeletal mineralization and vascular calcification, applying IALP to dephosphorylate phosphoproteins, e.g., OPN, should be interpreted with caution because of the delineated differences between BALP and IALP.

Whereas the majority of reports have described *Spp1* ^−/−^ mice (a.k.a., OPN knockout mice) as being largely normal, Fourier transform infrared imaging spectroscopy analysis has been used to document mineralization abnormalities in the *Spp1* ^−/−^ mice and to detect more mineral in the mutant animals than in wild-type controls [6, 40]. Ten-day-old *Spp1* ^−/−^ mice have more mineralized osteoid than wild-type controls, and in vitro cultures of calvarial osteoblasts produced more von Kossa-positive nodules over the course of a 21-day differentiation assay than wild-type controls [41]. The degree of severity of the hypermineralization phenotype in *Spp1* ^−/−^ mice is however very mild, a fact that we have attributed to the very high levels of extracellular PPi in these mice. At ten days of age, *Spp1* ^−/−^ mice have extracellular PPi levels even higher than those observed in the *Alpl* ^−/−^ mice, where extracellular PPi excess promotes rickets/osteomalacia [25]. We surmise that the increased levels of PPi compensate for the lack of OPN limiting what would otherwise be excessive mineralization due to the lack of OPN.

Complete removal of all phosphates bound to OPN is not possible with IALP or BALP but with TRAP. TRAP could, therefore, be a physiological regulator of OPNs’ inhibitory action on mineralization. The partial dephosphorylation of OPN achieved by BALP also reduces the inhibitory action of OPN and influences the mineralization. BALP is present at the site of mineralization and these data confirm that BALP also acts as a regulator of OPN [42]. Intriguingly, the present data suggest that several phosphorylations are implicated in the inhibitory action of OPN. Some of these phosphorylations, which are rapidly liberated by either BALP or TRAP, seem to be controlling approximately 40–50% of the inhibitory effect, while the remaining inhibition is among the last 18 phosphorylations only liberated by TRAP. This indicates that there are qualitative differences in the action of BALP and TRAP, which may have consequences for their functional roles in regulating mineralization. In addition, the different efficiencies in phosphate removal from OPN by TRAP and BALP may partly explain the observed heterogeneity in OPN phosphorylation in bone extracts [11, 43]. There are data indicating that the bone tissue of TRAP knockout mice (*Acp5* ^−/−^) is hypermineralized, as well as a disturbed mineralization in the growth plate [44]. However, these findings seem to be secondary to the reduced bone-resorptive activity of osteoclasts leading to a mild osteopetrotic phenotype. Moreover, inactivating mutations in the *ACP5* gene (encoding TRAP) cause the rare recessive disorder spondyloenchondrodysplasia, which includes autoimmunity disease features, e.g., systemic lupus erythematosus [45], associated with disturbed bone development and short stature probably mediated by impaired dephosphorylation of OPN [46, 47].

Besides partially dephosphorylating OPN, BALP is also an important mineralization promoter via its ability to hydrolyze the mineralization inhibitor PPi, thereby fine-tuning the local extracellular ratio between PPi and free phosphate pivotal for optimal mineralization conditions [3]. Considering the results from the current study, the question remains if BALP and TRAP co-regulate the inhibitory properties of OPN during mineralization. Narisawa et al. [42] found that OPN in long bones from *Alpl* ^−/−^ mice is hyperphosphorylated in comparison with WT (*Alpl* ^+/+^) control mice, while the degree of phosphorylation is decreased in OPN from *Alpl* ^−/−^ mice that overexpress human TNALP transgene in bone (i.e., *ColTg; Alpl* ^−/−^), which suggests that mouse TNALP and human TNALP dephosphorylate OPN in bone. Since BALP and TRAP are not co-expressed to a large extent in the same cells, or compartments in bone, it seems plausible that these enzymes mainly exert functions that are independent of each other.

It should be noted that the microenvironment, especially the pH at the site of action of these phosphatases, may considerably influence their action on the mineralization inhibitors PPi and OPN. Whereas BALP is active at basic pH, TRAP exerts optimal activity at acidic pH and show low activity at neutral and basic pH. It is possible that the action of TRAP on OPN and PPi may facilitate mineralization of bone remodeling units that occurs during the formation phase after the osteoclast has excavated the bone at acidic pH. It has been shown that TRAP remains associated to the resorbed bone after its secretion to the resorption pocket [22, 48], and might there modify the surface by, e.g., dephosphorylation of OPN to permit and facilitate subsequent bone formation in the resorption pit.

The current study has a number of strengths, including the use of BALP extracted from human bone tissue. Previous studies investigating the inhibitory properties of OPN have applied excessive amounts of IALP to dephosphorylate OPN. Needless to say, IALP is not present at the mineralization site, but more notably it is a different isozyme than BALP (TNALP expressed in bone) with approximately only 50% sequence identity [24]. Results from the present study demonstrate that BALP and IALP differ significantly in their dephosphorylating properties. In addition, this study presents the first data on the inhibitory effect of OPN dephosphorylated by TRAP on in vitro mineralization of human osteoblast-like cells. However, this study also possesses limitations that warrant consideration when interpreting the data. The degree of phosphorylation for OPN varies when isolated from different sources and species. Gericke et al. [49] investigated various forms of OPN (i.e., rat bone OPN, recombinant OPN, and bovine milk OPN), including different degrees of phosphorylation, and demonstrated the importance of phosphorylation as a pivotal factor in regulating OPN-mediated mineralization. We used bovine milk OPN with 28 phosphorylation sites in the current study, which inhibited the mineralization by approximately 70%.

To summarize, our results provide novel insight how both BALP and TRAP can alleviate the inhibitory effect of bovine milk OPN on mineralization. The current study indicates that both these phosphatases are important regulators of mineralization but with different roles in the mineralization process. The different roles may depend on site specificity, that is, typical osteoblast-derived mineralization by BALP, or mineralization facilitated by TRAP in the osteocyte lacunae and resorption pit to dephosphorylate newly released OPN which could inhibit de novo bone formation. This study presents evidence that TRAP displays enzymatic activity towards the endogenous substrates for BALP, i.e., PPi and PLP (in addition to OPN). BALP can dephosphorylate OPN partially and influence its inhibitory effect on mineralization, whereas TRAP can dephosphorylate OPN completely and eliminate the inhibitory effect of OPN. Further studies are needed to elucidate the possible physiological relevance of TRAP in the osteoblastic lineage, and the mechanistic interplay between BALP, TRAP, OPN, and PPi, during the intricate process of mineralization.
